# Synthesis, characterization and evaluation of antidengue activity of enantiomeric Schiff bases derived from S-substituted dithiocarbazate

**DOI:** 10.3906/kim-2006-22

**Published:** 2020-10-26

**Authors:** Maqsood MARYAM, Sang Loon TAN, Karen Ann CROUSE, Mohamed Ibrahim MOHAMED TAHIR, Hui-Yee CHEE

**Affiliations:** 1 Faculty of Natural Sciences, Sardar Bahadur Khan Women University, Balochistan, Quetta Pakistan; 2 Department of Medical Microbiology and Parasitology, Faculty of Medicine and Health Sciences, Universiti Putra Malaysia, SerdangSelangor Malaysia; 3 Department of Chemistry, Faculty of Science, Universiti Putra Malaysia, Serdang, Selangor Malaysia; 4 Research Centre for Crystalline Materials, School of Science and Technology, Sunway University, Sunway, Selangor Malaysia

**Keywords:** Dithiocarbazate Schiff base, enantiomers, camphor, camphorquinone, carvone, antidengue

## Abstract

A series of Schiff bases have been successfully synthesized through the acid-catalyzed condensation of S-substituted dithiocarbazates and three enantiomerically pure monoterpenes, (1 *R* )-(+)-camphor, (1 *S* )-(-)-camphor, (1 *R* )-(-)-camphorquinone, (1 *S* )-(+)-camphorquinone, ( *R* )-(-)-carvone and ( *S* )-(+)-carvone. Spectroscopic results revealed that the Schiff bases containing camphor or carvone likely adopted an *E* -configuration along the characteristic imine bond while those containing camphorquinone assumed a *Z* -configuration. The antidengue potential of these compounds was evaluated based on DENV 2 caused cytopathic effect (CPE) reduction-based in vitro evaluation. The compounds were validated through secondary foci forming unit reduction assay (FFURA). Compounds were also tested for their cytotoxicity against Vero cells. The compounds showed variable degrees of antiviral activity with the camphor compounds displaying the highest antidengue potential. The enantiomers of the compounds behaved almost similarly during the antiviral evaluation.

## 1. Introduction

Dengue virus (DENV) is a positive single-stranded RNA virus of the Flaviviridae family that causes dengue fever (DF) with or without warning signs, and severe dengue [1]. The four serotypes, DENV 1, DENV 2, DENV 3, and DENV 4, are genetically and antigenically distinct, and epidemiologically similar [2]. Infection with one serotype leads to all-time protection against homologous reinfection but only brief protection against heterologous types. DENV infection and immune system interactions may result in either immunopathology leading to severe forms of the disease, or recovery from infection [3]. DENV is one of the most serious arboviral threats in tropical and subtropical regions, with 50–100 million new cases annually among the 4 billion people in endemic regions [4]. It is spreading rapidly due to global demographic changes, rapid and unrestrained urbanization, population growth, and global ease of travel. WHO estimates that dengue has shown a 30-fold increase in detected cases globally over the past 5 decades [1]. There is no antiviral medication currently available for the treatment of dengue.

S-substituted dithiocarbazates and their Schiff base derivatives have received increasing attention over the years in the field of medicinal chemistry owing to their versatile functionalities to act as antimicrobial [5], anticancer [6], anthelmintic [7], antioxidative [8], anti-inflammatory [9], anticonvulsant, and antinociceptive agents [10], as well as nuclear medicines [11]. While the specific mode of action remains unclear, they are commonly believed to target metal containing active sites owing to the presence of hard and soft, nitrogen and sulfur donor atoms that can form stable chelating complexes and disrupt the physiological function of the target. Such a mechanism has been demonstrated by a closely related thiosemicarbazone Schiff base analog known as triapine, which was shown to be effective against different types of cancers and viruses in several clinical trials [12]. Dithiocarbazate Schiff base derivatives warrant further exploration since a wide variety of analogs can be derived by introducing different substituents into the structural framework potentially enriching their physico-chemical and biological activities.

**Scheme 1:**
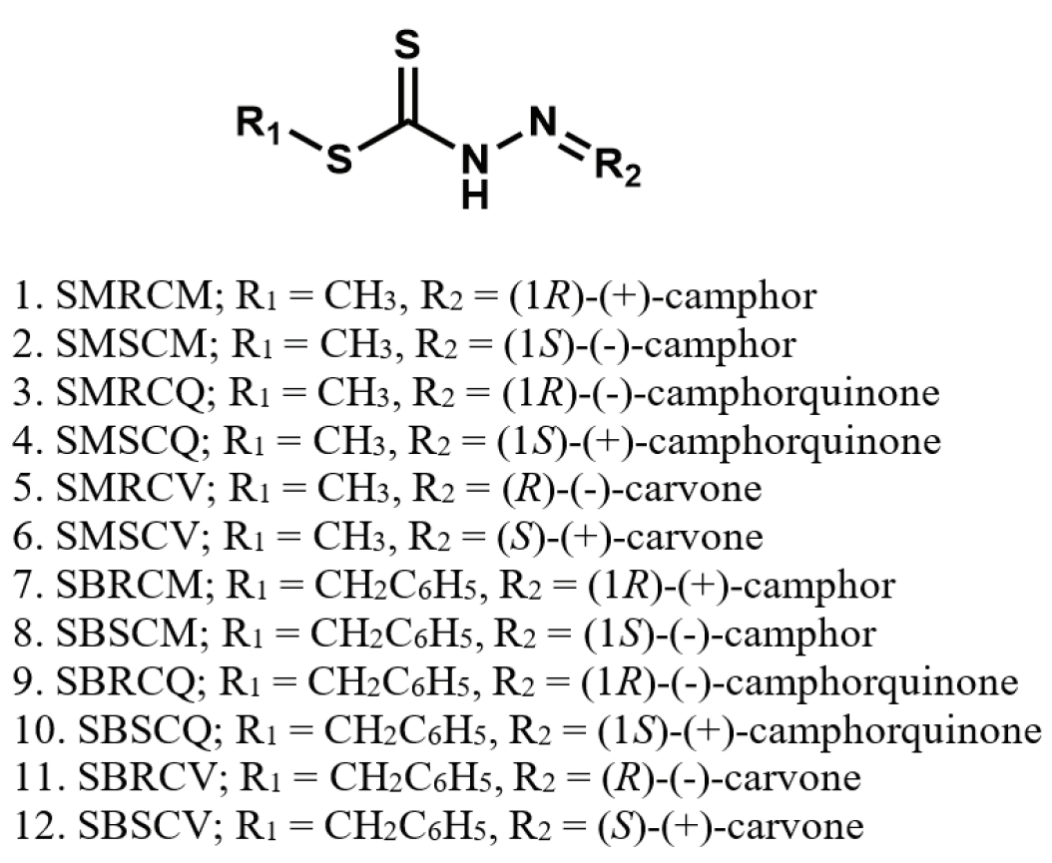
Synthesis of S-substituted dithiocarbazate Schiff bases.

There are no reports on evaluation of antidengue activity of dithiocarbazate derivatives. Research in the field has mainly focused on chemical extracts from medicinal plants and herbs due to their relatively low toxicity [13]. Among the vast number of natural products, terpenes and terpenoids represent a very important class of compounds due to their wide spectrum of biological activities including antimicrobial [14], anticancer [15], and antioxidant [16] characteristics. Recently, a few monoterpenes and terpenoids have been evaluated for the first time against dengue virus. They were found to be moderately active in inhibiting the replication of all four dengue serotypes in Vero Cells [17]. In view of the medicinal benefits of these compounds, we have embarked on the synthesis of a series of Schiff bases derived from S-methyl, S-benzyldithiocarbazates, and enantiomerically pure monoterpenes comprising (1 *R* )-(+)-camphor, (1 *S* )-(-)-camphor, (1 *R* )-(-)-camphorquinone, (1 *S* )-(+)-camphorquinone, ( *R* )-(-)-carvone, and ( *S* )-(+)-carvone (Scheme 1) to investigate whether such combinations would be active against dengue virus while demonstrating low toxicity towards the host cells.

## 2. Experimental

### 2.1. Materials and instrumentation

Unless otherwise mentioned, all chemical reagents were of ACS grade and were used as supplied without further purification. Melting points (m.p.) were measured without correction using a Barnstead-Electrothermal IA9100 digital melting point apparatus. IR spectra were recorded on a Perkin-Elmer FT-IR 100 system using attenuated total reflection (ATR) within the spectral range 4000–250 cm–1. Electronic spectra of 1 × 10–4 M solutions in DMSO were measured on a Shimadzu UV-2501PC spectrophotometer (250–800 nm). 1H and 13C{1H} NMR spectra were recorded on a JEOL JNM-ECA 400 NMR spectrometer (1H 400 MHz, 13C 100 MHz) with tetramethylsilane (TMS) being used as the internal standard and DMSO-d6 as the solvent. Mass spectra were obtained using a Shimadzu GCMS-QP5050A quadrupole mass spectrometer in electron ionization (EI) mode with direct insertion (DI). Carbon, hydrogen, and nitrogen analyses were performed on a LECO CHNS-932 analyzer under helium atmosphere with sulfamethazine being used as the standard. S-methyl (SMDTC) and S-benzyldithiocarbazates (SBDTC) were synthesized following reported procedures [18]. SMDTC (m.p. 354 K, 4.40 g, yield 72%); SBDTC (m.p. 398 K, 7.44 g, yield 75%).

### 2.2. Crystal structure determination

Diffraction data were collected at 150 K using an Enraf-Nonius Kappa CCD diffractometer with graphite-monochromatized Mo *K* α radiation (λ = 0.71073 Å). A multi-scan absorption correction was applied through DENZO-SMN package [19]. The structures were solved by dual-space algorithm and refined on *F* 2 by full matrix least-squares technique [20]with the anisotropic displacement parameters for all nonhydrogen atoms, of which the entire process was achieved through OLEX2 program [21]. The C-bound H atoms were placed at ideal geometrical position and refined in the riding model approximation with *U* iso(H) = 1.2–1.5 *U* eq (carrier atom). The N-bound H atoms were located from difference maps and refined with *U* iso(H) = 1.2 *U* eq(N). The absolute structure was determined based on differences in Friedel pairs included in the data set. The molecular structures and their packing diagrams were generated using ORTEP [22], and DIAMOND [23] respectively while the crystal data were analyzed by PLATON [24]. Details of unit cell data, X-ray data collection, and structure refinement are given in Table 1.

**Table 1:** Crystallographic data and refinement details for compounds 2, 4 and 5.

	2	4	5
Chemical formula	C12H20N2S2	C12H18N2OS2	C12H18N2S2
Mr	256.42	270.40	254.41
Crystal system	Orthorhombic	Monoclinic	Monoclinic
Space group	P 21 21 21	P 1 21 1	P 1 21 1
Temperature (K)	150	150	150
a (Å)	11.9408 (1)	6.9904 (2)	10.4610 (2)
b (Å)	12.6899 (2)	10.5192 (3)	12.6780 (2)
c (Å)	17.9505 (3)	9.5559 (2)	10.7956 (2)
α (°)	90	90	90
β (°)	90	101.2630 (1)	108.8900 (10)
γ (°)	90	90	90
V (Å3)	2720.00 (7)	689.15 (3)	1354.65 (4)
Z	8	2	4
Dcalc (g cm–3)	1.252	1.303	1.247
μ (MoKα, mm–1)	0.369	0.373	0.370
F(000)	1104	288	544
θmin, θmax (°)	5.112, 27.483	5.106, 27.488	5.127, 27.460
No. of reflections collected	6197	2789	5898
No. of independent reflections	6197	2789	5898
No. of reflections I > 2σ(I)	5414	2566	5215
Parameters refined	305	162	319
R1 [F2 > 2σ(F2)]	0.0362	0.0307	0.0366
wR(F2), all data	0.0890	0.0699	0.0875
S	1.058	1.054	1.053
Δρmax, Δρmin (eÅ-3)	0.239, –0.252	0.189, –0.183	0.211, –0.250

### 2.3. General procedure for the preparation of compounds 1–4 and 7–10

An equimolar amount of enantiomeric pure (1 *R* )-(+)-camphor (98%, Sigma-Aldrich Chemie GmbH, Steinheim, Germany; 1.52 g), (1 *S* )-(-)-camphor (99%, Sigma-Aldrich Chemie GmbH; 1.52 g), (1 *R* )-(-)-camphorquinone (99%, Sigma-Aldrich Chemie GmbH; 1.66 g), or (1 *S* )-(+)-camphorquinone (99%, Sigma-Aldrich Chemie GmbH; 1.66 g) was added to 0.01 mol, 1.22 g of SMDTC (or 0.01 mol, 1.98 g of SBDTC) dissolved in hot methanol (30 mL). Concentrated hydrochloric acid (37%, Merck KGaA, Darmstadt, Germany; 1.5 mL) was then added. The solution was reduced to half the initial volume and further heated for half an hour before being left to cool at ambient temperature. The product was filtered, washed with cold methanol, and recrystallized from methanol.

### 2.3.1.

Methyl (E)-2-((1R,4R)-1,7,7-trimethylbicyclo[2.2.1]heptan-2-ylidene)hydrazine-1-carbodithioate, SMRCM (1). White powder; yield 82%; m.p. 425–426 K; IR (ATR): = 3179 (m, νNH), 2966 (s, νCH), 2875 (w, νCH), 2828 (w, νCH), 1661 (m, νC=N), 1307 (s, νC–N), 1055 (s , νC=S), 963 (w, νN–N), 647 (m, νC–S) cm–1; 1H NMR (400 MHz, DMSO-d6): δ = 11.98 (1H, s, NH), 2.55 (2H, m, CH2), 2.44 (3H, s, CH3), 2.11 (1H, m, CH), 1.96 (2H, q, CH2, 3JHH = 4.58 Hz, 4JHH = 3.62 Hz), 1.25 (2H, m, CH2), 0.95 (3H, s, CH3), 0.90 (3H, s, CH3), 0.71 (3H, s, CH3) ppm; 13C{1H} NMR (100 MHz, DMSO-d6): δ = 198.24 (C=S), 170.82 (C=N), 52.89 (CN–C–CH3), 47.57 (CH3–C–CH3), 43.35 (CH2–CH–CH2), 35.10 (CN–CH2–CH), 32.21 (CN–C–CH2), 26.63 (CH2–CH–CH2), 19.18 (CH3–S), 18.43 (CH3–C–CH3), 16.79 (CH3–C–CH3), 11.07 (CN–C–CH3) ppm; UV/Vis (1×10–4 M, DMSO): λmax (ε) = 350 (394.5), 304 (20909), 273 (10783); MS (EI, 70 eV) m/z (%): 256 (8.2) [M]+, 183 (100); C12H20N2S2: (calc) C 56.21, H 7.86, N 10.92, (found) C 56.02, H 7.73, N 10.96.

### 2.3.2.

Methyl (E)-2-((1S,4S)-1,7,7-trimethylbicyclo[2.2.1]heptan-2-ylidene)hydrazine-1-carbodithioate, SMSCM (2). White powder; yield 80%; m.p. 425–426 K; ; IR (ATR): = 3177 (m, νNH), 2964 (s, νCH), 2877 (w, νCH), 2836 (w, νCH), 1660 (m, νC=N), 1303 (s, νC–N), 1047 (s , νC=S), 952 (w, νN–N), 636 (m, νC–S) cm–1; 1H NMR (400 MHz, DMSO-d6): δ = 11.98 (1H, s, NH), 2.55 (2H, m, CH2), 2.44 (3H, s, CH3), 2.11 (1H, m, CH), 1.96 (2H, q, CH2, 3JHH = 4.58 Hz, 4JHH = 3.62 Hz), 1.25 (2H, m, CH2) 0.95 (3H, s, CH3), 0.90 (3H, s, CH3), 0.71 (3H, s, CH3) ppm; 13C{1H} NMR (100 MHz, DMSO-d6): δ = 198.27 (C=S), 170.86 (C=N), 52.92 (CN–C–CH3), 47.61 (CH3–C–CH3), 43.38 (CH2–CH–CH2), 35.13 (CN–CH2–CH), 32.23 (CN–C–CH2), 26.66 (CH2–CH–CH2), 19.20 (CH3–S), 18.46 (CH3–C–CH3), 16.81 (CH3–C–CH3), 11.11 (CN–C–CH3) ppm; UV/Vis (1×10–4 M, DMSO): λmax (ε) = 350 (449), 304 (20981), 272 (10746); MS (EI, 70 eV) m/z (%): 256 (4.4) [M]+, 183 (100); C12H20N2S2: (calc.) C 56.21, H 7.86, N 10.92, (found) C 56.06, H 7.38, N 11.04.

### 2.3.3.

Methyl (Z)-2-((1S,4R)-4,7,7-trimethyl-3-oxobicyclo[2.2.1]heptan-2-ylidene)hydrazine-1-carbodithioate, SMRCQ (3). Yellow powder, yield 75%; m.p. 377–378 K; IR (ATR): = 3232 (m, νNH), 2958 (s νCH), 2942 (m, νCH), 2879 (w, νCH), 1713 (s, νC=O), 1607 (m, νC=N), 1263 (s, νC–N), 1088 (s , νC=S), 981 (w, νN–N), 657 (m, νC–S) cm–1; 1H NMR (400 MHz, DMSO-d6): δ = 12.74 (1H, s, NH), 3.51 (1H, t, CH, 3JHH = 4.6 Hz, 3JHH = 3.7 Hz), 2.50 (3H, s, CH3), 1.91 (2H, m, CH2), 1.42 (2H, m, CH2), 0.97 (3H, s, CH3), 0.93 (3H, s, CH3), 0.77 (3H, s, CH3) ppm; 13C{1H} NMR (100 MHz, DMSO-d6): δ = 204.53 (C=S), 203.02 (C=O), 154.75 (C=N), 57.94 (CO–C–CH3), 48.29 (CN–CH–CH2), 44.48 (CH3–C–CH3), 29.83 (CO–C–CH2), 23.75 (CN–CH–CH2), 20.37 (CH3–S), 17.40 (CH3–C–CH3), 17.24 (CH3–C–CH3), 9.03 (CO–C–CH3) ppm; UV/Vis (1×10–4 M, DMSO): λmax (ε) = 393.5 (11730), 332.5 (12097), 265.5 (5645); MS (EI, 70 eV) m/z (%): 270 (4.7) [M]+, 159 (100); C12H18N2OS2: (calc.) C 53.30, H 6.71, N 10.36, (found) C 52.17, H 6.64, N 10.93.

### 2.3.4.

Methyl (Z)-2-((1R,4S)-4,7,7-trimethyl-3-oxobicyclo[2.2.1]heptan-2-ylidene)hydrazine-1-carbodithioate, SMSCQ (4). Yellow powder; yield 75%; m.p. 377–378 K, IR (ATR): = 3244 (m, νNH), 2960 (s, νCH), 2942 (m, νCH), 2874 (w, νCH), 1713 (s, νC=O), 1606 (m, νC=N), 1265 (s, νC–N), 1088 (s , νC=S), 979 (w, νN–N), 655 (m, νC–S) cm–1; 1H NMR (400 MHz, DMSO-d6): δ = 12.75 (1H, s, NH), 3.51 (1H, t, CH, 3JHH=4.6 Hz, 3JHH=3.7 Hz), 2.50 (3H, s, CH3), 1.91 (2H, m, CH2), 1.43 (2H, m, CH2), 0.97 (3H, s, CH3), 0.94 (3H, s, CH3), 0.77 (3H, s, CH3) ppm; 13C{1H} NMR (100 MHz, DMSO-d6): δ = 204.55 (C=S), 203.03 (C=O), 154.77 (C=N), 57.96 (CO–C–CH3), 48.30 (CN–CH–CH2), 44.49 (CH3–C–CH3), 29.83 (CO–C–CH2), 23.76 (CN–CH–CH2), 20.39 (CH3–S), 17.41 (CH3–C–CH3), 17.27 (CH3–C–CH3), 9.05 (CO–C–CH3) ppm; UV/Vis (1×10–4 M, DMSO): λmax (ε) = 394.2 (12273), 333.0 (12846), 266.5 (6017); MS (EI, 70 eV) m/z (%): 270 (1.1) [M]+, 159 (100); C12H18N2OS2: (calc.) C 53.30, H 6.71, N 10.36, (found) C 51.87, H 6.52, N 10.27.

### 2.3.5.

Benzyl (E)-2-((1R,4R)-1,7,7-trimethylbicyclo[2.2.1]heptan-2-ylidene)hydrazine-1-carbodithioate, SBRCM (7). White powder; yield 85%; m.p. 387–388 K; IR (ATR): = 3189 (m, νNH), 2952 (m, νCH), 2900 (w, νCH), 1652 (m, νC=N), 1307 (s, νC–N), 1033 (s , νC=S), 963 (w, νN–N), 629 (m, νC–S) cm–1; 1H NMR (400 MHz, DMSO-d6): δ = 12.04 (1H, s, NH), 7.40 (2H, d, ortho-CH, 3JHH = 7.36 Hz), 7.32 (1H, m, para-CH), 7.28 (2H, t, meta-CH, 3JHH = 7.28 Hz), 4.42 (2H, s, S–CH2), 2.56 (2H, m, CH2), 2.12 (1H, m, CH), 1.95 (2H, q, CH2, 3JHH = 4.52 Hz, 3JHH = 3.70 Hz), 1.24 (2H, m, CH2), 0.90 (3H, s, CH3), 0.89 (3H, s, CH3), 0.70 (3H, s, CH3) ppm; 13C{1H} NMR (100 MHz, DMSO-d6): δ = 196.50 (C=S), 171.18 (C=N), 137.19 (ipso-C), 129.15 (2C, ortho-C), 128.51 (2C, meta-C), 127.10 (para-C), 52.94 (CN–C–CH3), 47.60 (CH3–C–CH3), 43.34 (CH2–CH–CH2), 37.55 (CH2–S), 35.16 (CN–CH2–CH), 32.18 (CN–C–CH2), 26.60 (CH2–CH–CH2), 19.18 (CH3–C–CH3), 18.40 (CH3–C–CH3), 11.07 (CN–C–CH3) ppm; UV/Vis (1×10–4 M, DMSO): λmax (ε) =346 (1454), 307 (18667), 275 (12502); MS (EI, 70 eV) m/z (%): 332 (0.8) [M]+, 91 (100); C18H24N2S2: (calc.) C 65.02, H 7.27, N 8.42, (found) C 64.49, H 7.14, N 8.23.

### 2.3.6.

Benzyl (E)-2-((1S,4S)-1,7,7-trimethylbicyclo[2.2.1]heptan-2-ylidene)hydrazine-1-carbodithioate, SBSCM (8). White powder; yield 85%; m.p. 387–388 K; IR (ATR): = 3192 (m, νNH), 2953 (m, νCH), 2900 (w, νCH), 1652 (m, νC=N), 1306 (s, νC–N), 1045 (s , νC=S), 958 (w, νN–N), 629 (m, νC–S) cm–1; 1H NMR (400 MHz, DMSO-d6): δ = 12.04 (1H, s, NH), 7.39 (2H, d, ortho-CH, 3JHH = 7.4 Hz), 7.32 (1H, m, para-CH), 7.29 (2H, t, meta-CH, 3JHH = 7.28 Hz), 4.42 (2H, s, S–CH2), 2.56 (2H, m, CH2), 2.11 (1H, m, CH), 1.96 (2H, q, CH2, 3JHH = 4.50 Hz, 3JHH = 3.68 Hz), 1.24 (2H, m, CH2), 0.90 (3H, s, CH3), 0.89 (3H, s, CH3), 0.70 (3H, s, CH3) ppm; 13C{1H} NMR (100 MHz, DMSO-d6): δ = 196.51 (C=S), 171.21 (C=N), 137.20 (ipso-C), 129.24 (2C, ortho-C), 128.47 (2C, meta-C), 127.12 (para-C), 52.97 (CN–C–CH3), 47.61 (CH3–C–CH3), 43.35 (CH2–CH–CH2), 37.56 (CH2–S), 35.18 (CN–CH2–CH), 32.20 (CN–C–CH2), 26.61 (CH2–CH–CH2), 19.19 (CH3–C–CH3), 18.42 (CH3–C–CH3), 11.10 (CN–C–CH3) ppm; UV/Vis (1×10–4 M, DMSO): λmax (ε) =346 (1418), 306 (18693), 276 (12507); MS (EI, 70 eV) m/z (%): 332 (3.2) [M]+, 91 (100); C18H24N2S2: (calc.) C 65.02, H 7.27, N 8.42, (found) C 64.17, H 7.03, N 8.26.

### 2.3.7.

Benzyl (Z)-2-((1S,4R)-4,7,7-trimethyl-3-oxobicyclo[2.2.1]heptan-2-ylidene)hydrazine-1-carbodithioate, SBRCQ (9). Yellow powder; yield 70%; m.p. 417–418 K; IR (ATR): = 3248 (m, νNH), 2964 (m, νCH), 2908 (w, νCH), 1707 (s, νC=O), 1605 (m, νC=N), 1268 (s, νC–N), 1062 (s , νC=S), 987 (w, νN–N), 660 (m, νC–S) cm–1; 1H NMR (400 MHz, DMSO-d6): δ = 12.81 (1H, s, NH), 7.40 (2H, d, ortho-CH, 3JHH = 7.36 Hz), 7.32 (1H, m, para-CH), 7.29 (2H, t, meta-CH, 3JHH = 7.20 Hz), 4.43 (2H, s, S–CH2), 3.50 (1H, t, 3JHH = 4.58 Hz, 3JHH = 3.71 Hz), 1.91 (2H, m, CH2), 1.42 (2H, m, CH2), 0.96 (3H, s, CH3), 0.92 (3H, s, CH3), 0.76 (3H, s, CH3) ppm; 13C{1H} NMR (100 MHz, DMSO-d6): δ = 204.49 (C=S), 201.11 (C=O), 154.94 (C=N), 136.45 (ipso-C), 129.18 (2C, ortho-C), 128.48 (2C, meta-C), 127.29 (para-C), 57.96 (CO–C–CH3), 48.34 (CN–CH–CH2), 44.50 (CH3–C–CH3), 37.95 (CH2–S), 29.82 (CO–C–CH2), 23.76 (CN–CH–CH2), 20.41 (CH3–C–CH3), 17.40 (CH3–C–CH3), 9.04 (CO–C–CH3) ppm; UV/Vis (1×10–4 M, DMSO): λmax (ε) =390.5 (5774), 330.5 (9275), 299.5 (7496), 266.5 (7570); MS (EI, 70 eV) m/z (%): 346 (21.7) [M]+, 91 (100); C18H22N2OS2: (calc.) C 62.39, H 6.4, N 8.08, (found) C 62.41, H 6.23, N 7.94.

### 2.3.8.

Benzyl (Z)-2-((1R,4S)-4,7,7-trimethyl-3-oxobicyclo[2.2.1]heptan-2-ylidene)hydrazine-1-carbodithioate, SBSCQ (10). Yellow powder; yield 75%; m.p. 417–418 K; IR (ATR): = 3247 (m, νNH), 2962 (m, νCH), 2901 (w, νCH), 1706 (s, νC=O), 1605 (m, νC=N), 1269 (s, νC–N), 1098 (s , νC=S), 994 (w, νN–N), 661 (m, νC–S) cm–1; 1H NMR (400 MHz, DMSO-d6): δ = 12.79 (1H, s, NH), 7.39 (2H, d, ortho-CH, 3JHH = 7.36 Hz), 7.32 (1H, m, para-CH), 7.29 (2H, t, meta-CH, 3JHH = 7.20 Hz), 4.44 (2H, s, S–CH2), 3.51 (1H, t, 3JHH = 4.58 Hz, 3JHH = 3.72 Hz), 1.92 (2H, m, CH2), 1.41 (2H, m, CH2), 0.92 (3H, s, CH3), 0.96 (3H, s, CH3), 0.76 (3H, s, CH3) ppm; 13C{1H} NMR (100 MHz, DMSO-d6): δ = 204.42 (C=S), 201.08 (C=O), 154.91 (C=N), 136.43 (ipso-C), 129.21 (2C, ortho-C), 128.44 (2C, meta-C), 127.24 (para-C), 57.91 (CO–C–CH3), 48.33 (CN–CH–CH2), 44.44 (CH3–C–CH3), 37.91 (CH2–S), 29.78 (CO–C–CH2), 23.72 (CN–CH–CH2), 20.37 (CH3–C–CH3), 17.36 (CH3–C–CH3), 8.99 (CO–C–CH3) ppm; UV/Vis (1×10–4 M, DMSO): λmax (ε) =389.5 (7241), 299.5 (12385), 300.5 (9276), 266.5 (8753); MS (EI, 70 eV) m/z (%): 346 (9.1) [M]+, 91 (100); C18H22N2OS2: (calc.) C 62.39, H 6.4, N 8.08, (found) C 61.48, H 6.17, N 8.15.

## 2.4. General procedure for the preparation of compounds 5–6 and 11–12

The synthesis was similar to the rest of the compounds except that 1 mL of diluted HCl (1 M) was used and the reaction was conducted at room temperature.

### 2.4.1.

Methyl (R,E)-2-(2-methyl-5-(prop-1-en-2-yl)cyclohex-2-en-1-ylidene)hydrazine-1-carbodithioate, SMRCV (5). Pale yellow powder; yield 87%; m.p. 391–393 K; IR (ATR): = 3155 (m, νNH), 2962 (m, νCH), 2925 (m, νCH), 2869 (w, νCH), 1640 (w, νC=N), 1587 (w, νC=C), 1326 (s, νC–N), 1061 (s , νC=S), 964 (w, νN–N), 632 (m, νC–S) cm–1; 1H NMR (400 MHz, DMSO-d6): δ = 12.34 (1H, s, NH), 6.29 (1H, s, CH), 4.78 (2H, d, CH2, 3JHH = 6.4 Hz), 3.02 (1H, m, CH), 2.44 (3H, s, CH3), 2.31 (2H, m, CH2), 2.10 (2H, m, CH2), 1.82 (3H, s, CH3), 1.73 (3H, s, CH3) ppm; 13C{1H} NMR (100 MHz, DMSO-d6): δ = 199.73 (C=S), 153.45 (C=N), 147.44 (CN–C=CH), 136.21 (CN–C=CH), 131.95 (CH–C=CH2), 110.21 (CH–C=CH2), 38.90 (CH–C=CH2), 30.23 (CH2–CH–CH2), 29.79 (CH2–CH–CH2), 20.74 (CH3–S), 17.54 (CN–C–CH3), 17.0 (CH–C–CH3) ppm; UV/Vis (1×10–4 M, DMSO): λmax (ε) = 340.5 (17786), 323.4 (21738), 278.1 (6194); MS (EI, 70 eV) m/z (%): 254 (36.8) [M]+, 91 (100); C12H18N2S2: (calc.) C 56.65, H 7.13, N 11.01, (found) C 55.43, H 7.21, N 10.04.

### 2.4.2.

Methyl (S,E)-2-(2-methyl-5-(prop-1-en-2-yl)cyclohex-2-en-1-ylidene)hydrazine-1-carbodithioate SMSCV (6). Pale yellow powder; yield 80%; m.p. 391–393 K; IR (ATR): = 3158 (m, νNH), 2973 (w, νCH), 2953 (m, νCH), 2922 (w, νCH), 2877 (w, νCH), 1640 (w, νC=N), 1585 (w, νC=C), 1325 (s, νC–N), 1061 (s , νC=S), 962 (w, νN–N), 637 (m, νC–S) cm–1; 1H NMR (400 MHz, DMSO-d6): δ = 12.34 (1H, s, NH), 6.29 (1H, s, CH), 4.78 (2H, d, CH2, 3JHH = 6.4 Hz), 3.02 (1H, m, CH), 2.44 (3H, s, CH3), 2.30 (2H, m, CH2), 2.10 (2H, m, CH2), 1.82 (3H, s, CH3), 1.73 (3H, s, CH3), ppm; 13C{1H} NMR (100 MHz, DMSO-d6): δ = 199.73 (C=S), 153.42 (C=N), 147.43 (CN–C=CH), 136.20 (CN–C=CH), 131.97 (CH–C=CH2), 110.21 (CH–C=CH2), 38.88 (CH–C=CH2), 30.23 (CH2–CH–CH2), 29.81 (CH2–CH–CH2), 20.73 (CH3–S), 17.54 (CN–C–CH3), 17.0 (CH–C–CH3) ppm; UV/Vis (1×10–4 M, DMSO): λmax (ε) = 341.2 (18367), 322.1 (22546), 279.5 (6827); MS (EI, 70 eV) m/z (%): 254 (57.0) [M]+, 91.1 (100); C12H18N2S2: (calc.) C 56.65, H 7.13, N 11.01, (found) C 55.37, H 7.02, N 10.87.

### 2.4.3.

Benzyl (R,E)-2-(2-methyl-5-(prop-1-en-2-yl)cyclohex-2-en-1-ylidene)hydrazine-1-carbodithioate SBRCV (11). Pale yellow powder; yield 65%; m.p. 405–406 K; IR (ATR): =3184 (m, νNH), 2921 (m, νCH), 2868 (w, νCH), 1642 (w, νC=N), 1588 (w, νC=C), 1315 (s, νC–N), 1060 (s , νC=S), 974 (w, νN–N), 634 (m, νC–S) cm–1; 1H NMR (400 MHz, DMSO-d6): δ = 12.42 (1H, s, NH), 7.39 (2H, d, ortho-CH, 3JHH = 7.32 Hz), 7.32 (1H, m, para-CH), 7.29 (2H, t, meta-CH, 3JHH = 7.16 Hz), 6.30 (1H, s, CH), 4.80 (2H, d, CH2, 3JHH = 6.4 Hz), 4.44 (2H, s, S–CH2), 3.04 (1H, m, CH), 2.32 (2H, m, CH2), 2.12 (2H, m, CH2), 1.79 (3H, s, CH3), 1.75 (3H, s, CH3) ppm; 13C{1H} NMR (100 MHz, DMSO-d6): δ = 197.79 (C=S), 153.77 (C=N), 147.32 (CN–C=CH), 137.12 (ipso-C), 136.38 (CN–C=CH), 131.79 (CH–C=CH2), 129.17 (2C, ortho-C), 128.42 (2C, meta-C), 127.08 (para-C), 110.11 (CH–C=CH2), 39.99 (CH–C=CH2), 37.57 (CH2–S), 30.19 (CH2–CH–CH2), 29.69 (CH2–CH–CH2), 20.64 (CN–C–CH3), 17.43 (CH–C–CH3) ppm; UV/Vis (1×10–4 M, DMSO): λmax (ε) =327.0 (20536), 287.0 (10013); MS (EI, 70 eV) m/z (%): 330 (7.3) [M]+,91 (100); C18H22N2S2: (calc.) C 65.41, H 6.71, N 8.48, (found) C 65.45, H 6.54, N 9.54.

### 2.4.4.

Benzyl (S,E)-2-(2-methyl-5-(prop-1-en-2-yl)cyclohex-2-en-1-ylidene)hydrazine-1-carbodithioate SBSCV (12). Pale yellow powder; yield 68%; m.p. 405–406 K; IR (ATR): = 3189 (m, νNH), 2924 (m, νCH), 2860 (w, νCH), 1643 (w, νC=N), 1585 (w, νC=C), 1314 (s, νC–N), 1059 (s , νC=S), 970 (w, νN–N), 634 (m, νC–S) cm–1; 1H NMR (400 MHz, DMSO-d6): δ = 12.40 (1H, s, NH), 7.39 (2H, d, ortho-CH, 3JHH = 7.32 Hz), 7.32 (1H, m, para-CH), 7.29 (2H, t, meta-CH, 3JHH = 7.18 Hz), 6.30 (1H, s, CH), 4.80 (2H, d, CH2, 3JHH = 6.4 Hz,), 4.45 (2H, s, S–CH2), 3.04 (1H, m, CH), 2.32 (2H, m, CH2), 2.12 (2H, m, CH2), 1.80 (3H, s, CH3), 1.76 (3H, s, CH3) ppm; 13C{1H} NMR (100 MHz, DMSO-d6): δ = 197.79 (C=S), 153.77 (C=N), 147.32 (CN–C=CH), 137.12 (ipso-C), 136.26 (CN–C=CH), 131.83 (CH–C=CH2), 129.16 (2C, ortho-C), 128.55 (2C, meta-C), 127.17 (para-C), 110.28 (CH–C=CH2), 40.19 (CH–C=CH2), 37.64 (CH2–S), 30.20 (CH2–CH–CH2), 29.01 (CH2–CH–CH2), 20.52 (CN–C–CH3), 17.54 (CH–C–CH3) ppm; UV/Vis (1×10–4 M, DMSO): λmax (ε) =330.2 (8724), 290.8 (9812); MS (DI, 70 eV) m/z (%): 330 (3.6) [M]+,91 (100); C18H22N2S2: (calc.) C 65.41, H 6.71, N 8.48, (found) C 63.73, H 6.54, N 8.23.

## 2.5. Virus and cells for antiviral evaluation

Aedes albopictus clone C6/36 cells were maintained to propagate the dengue virus type 2 (DENV 2); African green monkey ( *Chlorocebus* sp.) kidney Vero cells were grown for antiviral analysis. Both cells were grown in Eagle’s Minimum Essential Medium (EMEM) (Biowest LLC, Riverside, MO, USA) with 10% Fetal Bovine Serum (FBS) (Biowest LLC).

Ribavirin tablets (Copegus, 200 mg, Genentech, Inc., South San Francisco, CA, USA) were purchased and stock solutions were stored. Dilutions were prepared in 2% FBS (maintenance media) at the time of assay. TCID50 assay was employed to measure the titer of the virus stocks for antiviral evaluation following standard methods [25]. The viral CPE was observed on the third and fourth days and observations were recorded based on the Reed–Muench method.

### 2.5.1. MTT cytotoxicity assay

The 96-well plates were seeded with 100 µL of Vero cell suspension at a density of 1 × 105 cells / mL and the next day were treated with different dilutions of the synthesized compounds. Control wells were treated with only the vehicle media used to prepare the antiviral dilutions. Plates were incubated for four days at 37 °C. Following the addition of 15 μL of MTT (Sigma-Aldrich Corp., St. Louis, MO, USA) solution into each well, the plates were incubated at 37 oC for another 4 h. Subsequently, the medium was carefully aspirated from all the wells and 100 μL of DMSO was added to each well [26]. The optical density (OD) of the wells was measured at 570 nm using a 96-well plate reader (Tecan Group AG, Männedorf, Switzerland). The percentage cell viability was calculated against the untreated controls. Dose-response curves were plotted. Half maximal cytotoxic concentration (CC50) was calculated using Graph Pad Prism for Windows, Version 5 (GraphPad Software Inc., San Diego, CA, USA).

### 2.5.2. CPE reduction-based antiviral assay

Plates were seeded with Vero cell suspension (100 µL) at a density of 1 × 105 cells/mL one day before the wells were treated with TCID50 (100 µL) dilution of the DENV 2 stock and untreated control wells were maintained in parallel. After 1 h of viral adsorption, unbound viruses were washed and different antiviral dilutions were added. These plates were further incubated for 4 more days and were observed daily [26,27]. Observations were recorded on the third and fourth days. The wells were marked for CPE reduction according to the grading system defined by Kudi and Myint [28].

### 2.5.3. Foci forming unit reduction assay (FFURA)

Antiviral activity was quantitatively validated by measuring the reduction in the number of DENV infectious foci after 4 days of treatment. DENV 2 infected Vero cells were treated with different antiviral dilutions supplemented with 2% FBS and 1.5% carboxymethyl cellulose (CMC). Virus foci were stained and visualized according to the published protocol [29]. The number of foci was counted through stereomicroscope and virus titer was stated as foci forming unit (FFU). To calculate the antiviral activity of antidengue compounds, the percentage reduction in foci (%RF) was calculated between the treated, and untreated wells maintained in parallel. The assay was repeated three times, dose-response curves were plotted and half minimal inhibitory concentration (IC50) and selectivity index (SI) were calculated.

## 3. Results and discussion

### 3.1. Chemistry

#### 3.1.1. Synthesis and spectroscopic characterization of compounds 1–4, and 7–10

Conventionally, the formation of Schiff bases involving SMDTC or SBDTC is rather facile without much reliance on factors such as temperature, pH, and catalytic reagents [30]. In this study, however, the condensation reaction did not take place even after prolonged reflux at elevated temperature. A plausible reason for the failure of the reaction is that the structurally rigid bicyclic ring of the monoterpenes (camphor and camphorquinone in particular) coupled with the weakened dipole moment and reduced electrophilicity of the carbonyl carbon might have inhibited the reactivity by preventing nucleophilic attack from the electronegative S-substituted dithiocarbazates. One possible way to circumvent the inertness of the reaction is through capitalization of the electro negativity of the carbonyl oxygen by introducing a Lewis acid such as hydrogen ion to activate the nucleophilic oxygen.

IR analysis shows the presence of some essential peaks at about 3150–3250 cm–1 and 1605–1660 cm–1 which can be attributed to secondary νN–H and νC=N peaks. The difference between the νN–H peaks and the two distinctive peaks characteristic of asymmetric and symmetric stretching of primary amines as observed for SM- and SBDTC, as well as the absence of νC = N in the spectra of the precursors imply that condensation has indeed taken place between the dithiocarbazates and monoterpenes. While the disappearance of νC=O frequency in SMRCM, SMSCM, SBRCM, and SBSCM is further evidence which confirms the formation of the Schiff bases, the peak is still observed at 1706–1713 cm–1 in the corresponding spectra of SMRCQ, SMSCQ, SBRCQ, and SBSCQ and it is more resolved compared to the broad asymmetric C=O peak of the starting precursor (i.e. camphorquinone) at ca. 1745 cm–1. This suggests that only one of the C = O groups reacted with the substituted dithiocarbazates, while the other C=O which is likely the one adjacent to the methyl group, remains intact due to steric hindrance. νC=S at 1030–1100 cm–1 together with the absence of νS–H (2600–2700 cm–1) are clear evidence that the solid Schiff bases are predominantly in their thione tautomeric form [31].

**Figure 1:**
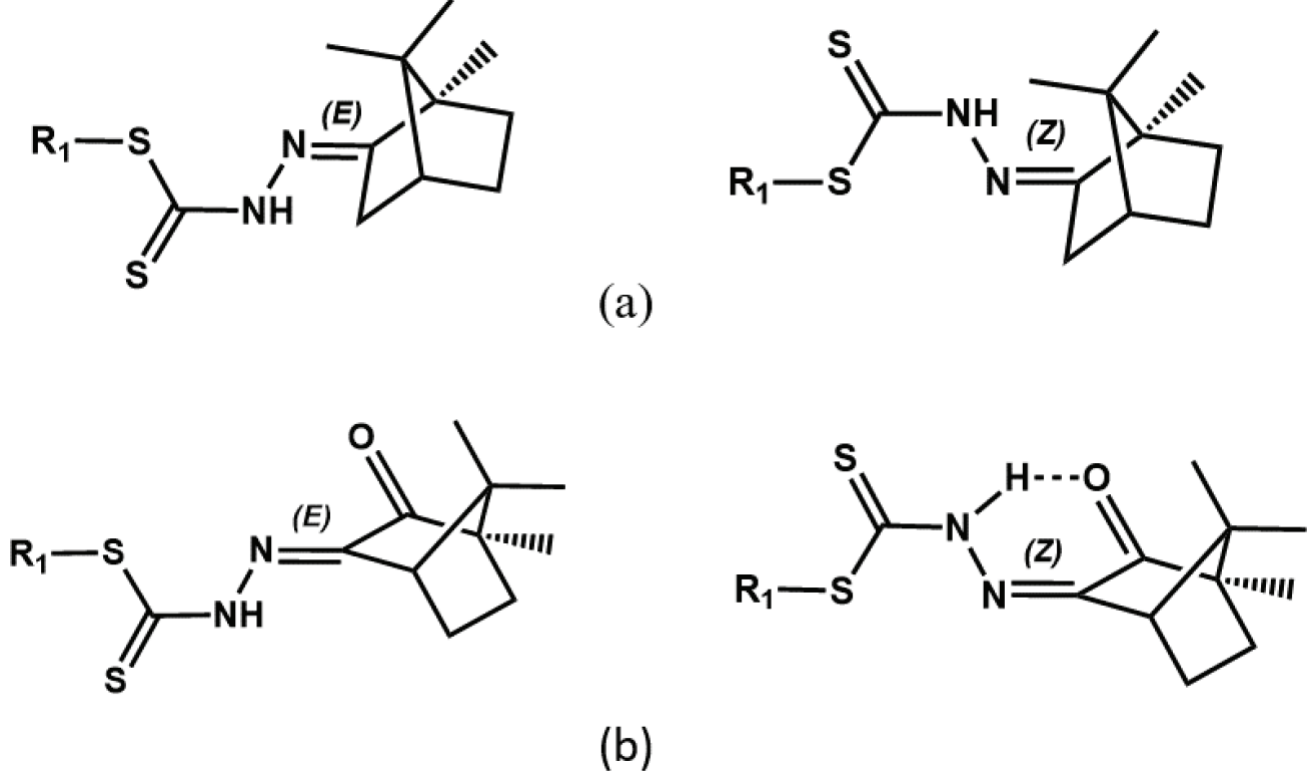
The EZ-configuration for (a) compounds 1, 2, 5 and 6 with all compounds primarily appearing in the E-configuration, (b) compounds 3, 4, 7 and 8 with all mainly appearing in the Z-configuration.

1H NMR spectra in DMSO-d6 show thionamide N–H peaks for SMRCM, SMSCM, SBRCM, and SBSCM at 11.98–12.04 ppm, which is within the typical range for *E* -configuration along the C=N imine bond [32]. SMRCQ, SMSCQ, SBRCQ, and SBSCQ Schiff bases exhibit a resonance for the thionamide proton between 12.71–12.81 ppm indicating *Z* -configuration (Figure 1a). Such configuration enables an intramolecular interaction between thionamide, and unreacted carbonyl groups resulting in the relatively high field chemical shift for the thionamide proton (Figure 1b). As expected, all Schiff bases remain in the thione tautomeric form even in a polar solvent like DMSO with no signal assignable to thiol proton being seen near 4 ppm [32]. The thione form of a dithiocarbazate Schiff base has been found to be more stable than its thiol counterpart by 14.5 kJ/mol through gas phase DFT calculation [33]. For the rest of the protons and carbon nuclei, their resonances appear in the typical range, and assignment has been unambiguously determined upon comparison with the 1H and 13C{1H} NMR spectra of the corresponding precursors (cf. Section 2.3. and 2.4.).

#### 3.1.2. Synthesis and spectroscopic characterization of compounds 5–6, and 11–12

**Scheme 2:**
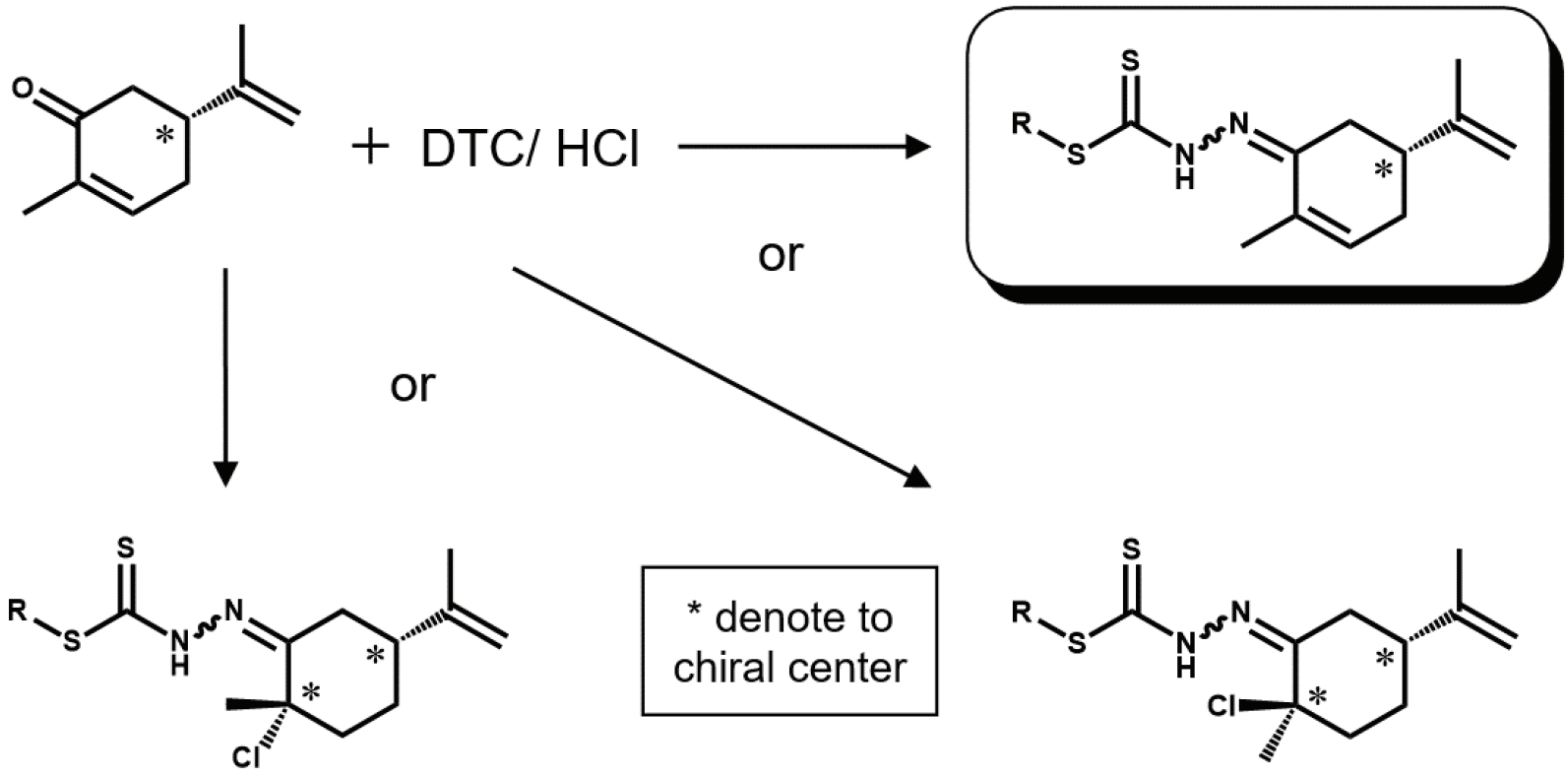
Possible outcomes of acid catalyzed condensation between carvone and substituted dithiocarbazates.

Since the enantiomeric carvones did not react with the substituted dithiocarbazates under the heat-and-reflux routine commonly used, acid was used to catalyze the reaction. However, the additional alkene functional group in carvone makes it susceptible to acid addition leading to undesired stereoisomerization or racemization of the enantiomerically pure reagents (see Scheme 2).

To reduce the possibility of the occurrence of the undesired side reactions, a catalytic amount of dilute acid was used and, in addition, the synthesis was conducted at room temperature even though the condensation reaction, being an endothermic process, would have been favored by higher temperatures [34]. IR spectra of the carvone-DTC Schiff bases possess a profile similar to that of their camphor- and camphorquinone-DTC counterparts implying that the Schiff bases were successfully synthesized through acid-catalyzed condensation. More importantly, the presence of ν(C=C) at ca. 1582 cm–1 together with the absence of an intense ν(C-Cl) peak (~700 cm–1) prove that the acid addition reaction did not take place.

**Figure 2:**
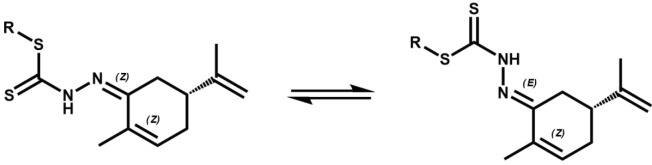
The EZ-configuration for compounds 5, 6, 11, and 12.

The thionamide protons in these compounds exhibit a resonance between 12.38 and 12.43 ppm which is close to those observed for the camphor-DTC Schiff bases indicating that they too adopt an *E* -configuration (Figure 2) along the C=N bond [32]. However, given that the substituted dithiocarbazate fragment is sterically less hindered about the N–N single bond compared to the bulkier camphor-DTC Schiff bases, the possibility of the formation of the *Z* -configuration, generated by flipping towards either side of the monocyclic ring of carvone, cannot be totally omitted. These compounds also display no sign of thione-thiol tautomerism in DMSO solution. The intactness of the alkene groups in the Schiff bases is confirmed by the 13C{1H} resonances at 136.34–148.00 ppm that are very close to those observed for the carvone precursors (142.70–143.82 ppm). In addition, there is no trace of any resonance signal for C-Cl at 50–75 ppm which would be expected if addition reaction occurred at the C=C.

The supplementary materials include the IR, EI-MS, 1H, and 13C{1H} NMR Spectra. Supplementary materials Figures S1–S3 are the IR and EI-MS spectra of compounds SMRCM, SMSCM, SBRCM, and SBSCM while, Figures S4–S11 are the 1H and 13C{1H} NMR Spectra of compounds SMRCM, SMSCM, SBRCM, and SBSCM. Figures S12–S14 are the IR and EI-MS spectra of compounds SMRCQ, SMSCQ, SBRCQ, and SBSCQ while, Figures S15–S22 are the 1H and 13C{1H} NMR Spectra of compounds SMRCQ, SMSCQ, SBRCQ, and SBSCQ. Figures S23–S25 are the IR and EI-MS spectra of compounds SMRCV, SMSCV, SBRCV, and SMSCV while, Figures S26–S33 are the 1H and 13C{1H} NMR Spectra of compounds SMRCV, SMSCV, SBRCV, and SMSCV.

### 3.2. Crystal structures of SMSCM (2), SMSCQ (4) and SMRCV (5)

**Figure 3:**
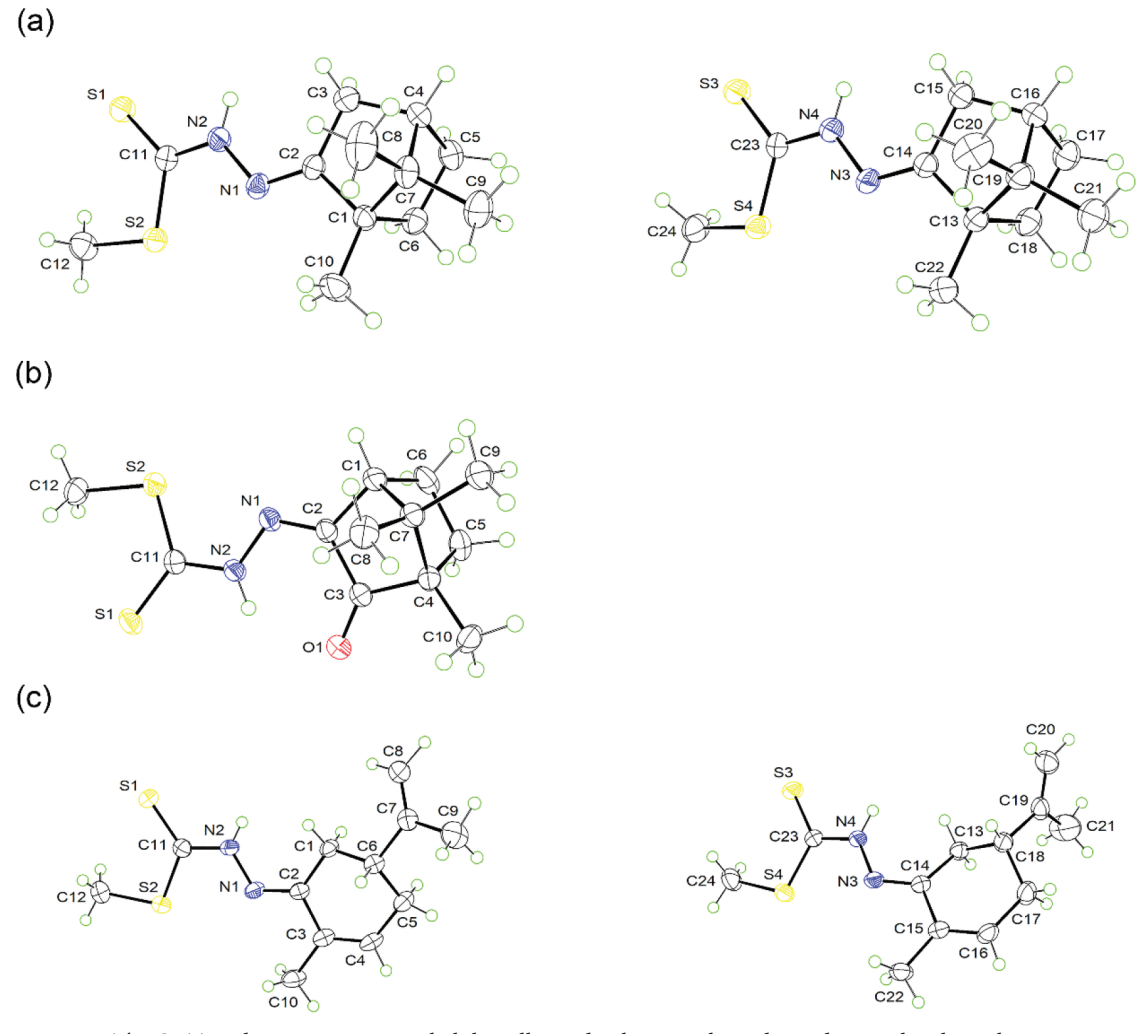
The ORTEP diagram at 50% probability ellipsoids, showing the independent molecule in the asymmetry unit for (a) SMSCM (2), (b) SMSCQ (4) and (c) SMRCV (5).

Figure 3 shows the ORTEP diagrams of SMSCM, SMSCQ, and SMRCV in which SMSCM (Figure 3a) comprises two molecules while SMSCQ (Figure 3b) contains only a single molecule and SMRCV (Figure 3c) comprises two molecules in the asymmetric unit. Each of the structures constituted of three distinct fragments, i.e. the dithiocarbazate (S2CN2), the appending methyl, and isocyclic ring. The central dithiocarbazate fragment for all structures is essentially planar as shown through the corresponding least-square plane fitting with a r.m.s. deviation of 0.0064 Å and 0.0123 Å for molecule I [S2–C11(S1)–N2–N1] and molecule II [S4–C23(S3)–N4–N3] in SMSCM, 0.0447 Å for SMSCQ as well as 0.0154 Å and 0.0347 Å for molecule I and II in SMRCV. Molecules I and II in SMSCM are slightly differed from each other in that the thiomethyl plane (S1–C11–S2–C12) of the former is relatively less twisted from the central dithiocarbazate plane [S2–C11(S1)–N2–N1] with a dihedral angle of 3.6(1)° as compared to 5.8(1)° for the equivalent planes [S3–C23–S4–C24// S4–C23(S3)–N4–N3] in molecule II. The superimposition of the two molecules results in a r.m.s. deviation of merely 0.073 Å. As for SMRCV, molecules I and II are relatively more deviated from each other with a r.m.s. deviation of the superimposed structures being 0.796 Å. Overall, the least-square plane of thiomethyl (S1–C11–S2–C12 or S3–C23–S4–C24) and isocylic ring (C1–C2–C3–C4–C5–C6 or C13–C14–C15–C16–C17–C18) are twisted from the central dithiocarbazate plane by 14.7(1)° for molecule I and 10.7(1)° for molecule II, respectively.

As consistent with the spectroscopic findings, the solid-state structure of SMSCM and SMRCV appear in *E* –configuration along the C = N imine bond with the torsion angle of –177.5(2) and –176.9(2)° across N2–N1–C2–C1 and N4–N3–C14–C13 for SMSCM as well as –179.1(2) and 178.3(2)° across N2–N1–C2–C3 and N4–N3–C14–C15 for SMRCV, and the same is true for SMSCQ which exists as *Z–* configuration with the torsion angle for N2–N1–C2–C3 being –1.0(4)°. The bond length of C11–S1 (or C23–S3 in the second molecule) for all structures is relatively shorter than C11–S2 or C23–S4 (cf. geometry parameters in Table 2) which reflects the thione character of those DTC Schiff bases. The azine (N2–N1, and N4–N3), and methyl (C12 or C24) residues are respectively arranged in the trans and cis position with respect to thione group (C11=S1 or C23=S3), hence constitute a trans-cis conformation as observed in the majority of DTC Schiff bases in the Cambridge Structural Database [35], with the torsion angles across S1–C11–N2–N1 (or S3–C23–N3–N4), and S1–C11–S2–C12 (or S3–C23–S4–C24) ranged between 171.7(2)°–179.7(1)° and 1.7(3)°–6.8(2)° correspondingly.

**Figure 4:**
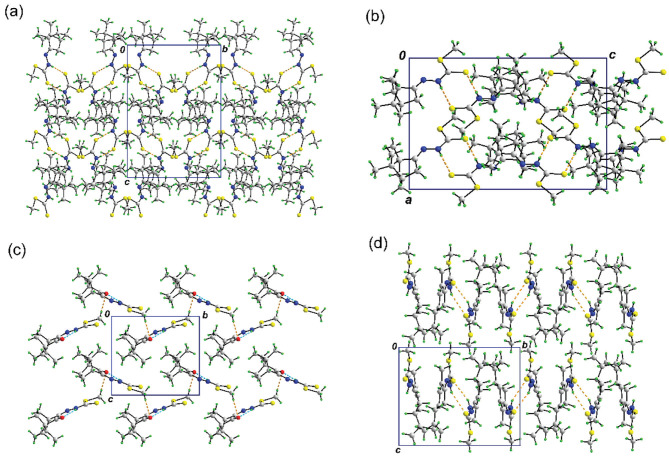
(a) Molecular packing of SMSCM sustained by pairwise N–H···S interaction leading to the formation of eight-membered {···NHCS}2 homosynthon related by 21 symmetry as view along the crystallographic a-direction, (b) a projected view along the b-direction, showing the 21-symmetry related molecular packing of SMSCM, (c) molecular packing of SMSCQ governed by intermolecular C–H···O (orange dashed line) and intramolecular N–H···O (blue dashed line) interactions to form a CH···O···HN heterosynthon arranged in a zigzag array along the b-direction, and (d) molecular packing of SMRCV associated by eight-membered {···NHCS}2 homosynthon replicated by 21 symmetry along the crystallographic a direction.

In the terms of supramolecular features, the crystal packing of SMSCM and SMRCV are mainly governed by pairwise N–H···S interactions between the molecules in the asymmetric unit leading to the formation of an eight-membered {···NHCS}2 homosynthon which is replicated by 21 symmetry along all crystallographic directions for the former and along the *b* -plane for the latter, with both showing no directional interactions between those pairs of molecules (Figure 4a). In contrast, SMSCQ is sustained by C–H···O intermolecular interaction between C12–H12A···O1 together with an intramolecular interaction between N2–H2N···O1 leading to CH···O···HN heterosynthon that connects the molecules in a zigzag array, and extends along the *b* -direction (Figure 4b). The molecular packing of SMSCQ were governed by intermolecular C–H···O and intramolecular N–H···O interactions to form a CH···O···HN heterosynthon arranged in a zigzag array along the *b* -direction (Figure 4c). The molecular packing of SMRCV were associated by eight-membered {···NHCS}2 homosynthon replicated by 21 symmetry along the crystallographic *a* direction (Figure 4d). The stark difference observed in the crystal packing of SMSCQ as compared to SMSCM, or SMRCV is presumably owing to its arrangement in *Z* -configuration which is stabilized by intramolecular N–H···O interaction. It is noteworthy that N–H···O possesses greater interaction energy than N–H···S interaction and hence it might have prevented the formation of the typical eight-membered {···NHCS}2 homosynthon as observed in SMSCM, and SMRCV [36]. The geometric parameters characterizing the interactions for the corresponding structures are presented in Table 3.

**Table 2:** Selected geometric parameters (Å, °) for compounds 2, 4 and 5.

Parameter	2	4	5
N1–C2	1.277(3)	1.287(3)	1.295(4)
N1–N2	1.395(3)	1.369(3)	1.380(3)
N2–C11	1.340(3)	1.360(3)	1.344(4)
C11–S1	1.663(3)	1.653(3)	1.663(3)
C11–S2	1.762(3)	1.749(2)	1.749(3)
S2–C12	1.795(3)	1.801(3)	1.797(3)
N3–C14	1.273(3)	-	1.285(4)
N3–N4	1.397(3)	-	1.391(3)
N4–C23	1.347(3)	-	1.334(4)
C23–S3	1.668(3)	-	1.672(3)
C23–S4	1.749(3)	-	1.746(3)
S4–C24	1.797(3)	-	1.794(3)
N2–N1–C2	117.3(2)	116.7(2)	118.6(2)
N1–N2–C11	118.7(2)	119.7(2)	118.6(2)
N2–C11–S1	122.0(2)	120.24(19)	121.8(2)
N2–C11–S2	112.96(19)	113.3(2)	112.9(2)
S1–C11–S2	125.04(15)	126.43(17)	125.30(18)
N4–N3–C14	116.5(2)	-	117.5(2)
N3–N4–C23	118.5(2)	-	119.3(2)
N4–C23–S3	121.1(2)	-	121.1(2)
N4–C23–S4	113.6(2)	-	113.4(2)
S3–C23–S4	125.30(16)	-	125.55(17)

**Table 3:** Hydrogen-bond geometry (Å, °) for compounds 2, 4 and 5.

D—H···A	D—H	H···A	D···A	D—H···A	Symmetry operation
2					
N2—H2N···S3	0.88(3)	2.69(3)	3.557(2)	173(3)	1-x, -½ + y, ½-z
N4—H4N···S1	0.87(4)	2.73(4)	3.602(3)	177(3)	1-x, ½ + y, ½-z
4					
C12—H12A···O1	0.96	2.56	3.516(4)	178	1-x, ½ + y, 1-z
N2—H2N···O1	0.86(4)	2.15(3)	2.801(3)	132(3)	
5					
N2—H2N···S3	0.79(4)	2.71(4)	3.459(3)	159(3)	x, y, 1 + z
N4—H4N···S1	0.90(4)	2.53(5)	3.369(2)	156(4)	1-x, ½ + y, ½-z

### 3.3. Biological assays

#### 3.2.1. MTT-based cytotoxicity and CPE reduction-based primary antiviral evaluation

The compounds did not display strong cytotoxicity. Toxicity decreased in the order carvone (CV) > camphorquinone (CQ) > camphor (CM). All twelve synthetic compounds showed a gradually decreasing trend in CC50 doses (Table 4).

The chirality in the form of R and S enantiomers did not show any differential effects on either the cytotoxic dose, or the antiviral activity. The two enantiomers of the compounds showed parallel antidengue potential. Therefore, they may have same pharmacodynamics and can be equally potent. If this is so, the compounds could be used in their enantiomerically pure forms or as a racemic mixture [37]. However, pharmacokinetics of these enantiomers requires further exploration.

The CM compounds showed higher antidengue activity than the other substituted Schiff bases whereas; the CQ and CV compounds were rather more toxic than the CM compounds (Table 2). The DTC-derivatives showed antibiotic potential in a study where some preexisting antibacterial molecules demonstrated greatly enhanced biological activity when conjugated with dithiocarbazate (DTC) [38]. During the present study, camphor compounds showed strong antidengue potential. Camphor is a terpenoid class of biologically active compounds found in the majority of natural products [39]. Camphor is a major component of many essential oils and the biological activities of essential oils are evident [40]. See supplementary materials Figure S34 for dose-response curves and Figures S35a–S35c for CPE-reduction during primary antiviral evaluation.

**Table 4:** CC50 doses and degree of CPE reduction in DENV 2-infected Vero cells upon treatment with synthetic Schiff base compounds.

No	Sample ID	M.W	CC50 (µM)	CPE reduction*
1.	SMRCM	256.4	88.4 ± 3.8	++
2.	SMSCM	256.4	88.7 ± 2.0	++
3	SMRCQ	270.4	65.2 ± 4.4	+
4	SMSCQ	270.4	69.2 ± 5.9	+
5	SMRCV	256.4	31.1 ± 8.0	--
6	SMSCV	256.4	24.5 ± 9.2	--
7	SBRCM	332.5	81.7 ± 4.4	++
8	SBSCM	332.5	87.2 ± 5.9	++
9	SBRCQ	346.5	58.4 ± 2.9	--
10	SBSCQ	346.5	58.7 ± 5.1	--
11	SBRCV	332.5	28.5 ± 3.9	+
12	SBSCV	332.5	29.2 ± 6.4	+

* DENV 2 CPE reduction caused by the compound treatments was marked as ++++ for total reduction, +++ for 75% reduction, ++ for 50% reduction, + for less than 50% reduction and -- for no reduction.

#### 3.2.2. Foci forming unit reduction (FFUR) based secondary antiviral evaluation

The treatment of Vero cells infected with DENV 2 with three different dilutions of the synthetic compounds revealed reduction in the number of foci appearance for only six of the synthesized compounds, SMRCM, SMSCM, SBRCM, SBSCM, SMRCQ, and SMSCQ. Similarly, the CC50 doses of these compounds (Table 4) were also lower than the six compounds showing viral inhibition during secondary evaluation. This may be due to the cytotoxicity of the compounds during viral foci development that may damage the cells before viral infection [41]. The treatments were compared to the nontreated virus infections incubated parallel. The six potential antiviral compounds were inhibiting the virus in almost a close range of concentration, around 20 µM of the compounds were able to inhibit the virus just about 100%.

The secondary evaluation provided half minimal inhibitory concentrations (IC50) for the six potential antiviral compounds between 6.2 µM and 7.8 µM. Selectivity index (SI) identified the window between the cytotoxic and inhibitory concentrations (Table 5). The SI values were within the range of 8.3 to 14.3, which are very close to each other, therefore, compounds can be considered equally potent. These six compounds were enantiomers of each other and the pattern of inhibition was very similar for each enantiomer of these compounds demonstrating that the antidengue activity might not be the virtue of the chirality of the compounds. See supplementary material, Figure S36 for antidengue effects of ribavirin as a positive control while, and Figures S37a and S37b for the antiviral effects of compounds during secondary evaluation.

**Table 5:** Selectivity index (SI) calculation for the six-potential in vitro antidengue Schiff bases identified after secondary evaluation.

No	Sample ID	CC50 ± SE(µM)	IC50 ± SE(µM)	Selectivity indexCC50/IC50
1.	SMRCM	88.4 ± 3.8	6.9 ± 1.2	12.8
2.	SMSCM	88.7 ± 2.0	6.2 ± 3.2	14.3
3.	SMRCQ	65.2 ± 4.4	7.8 ± 3.7	8.3
4.	SMSCQ	69.2 ± 5.9	6.8 ± 2.8	14.1
5.	SBRCM	81.7 ± 4.4	7.8 ± 4.5	10.4
6.	SBSCM	87.2 ± 5.9	6.8 ± 2.3	12.8

## 4. Conclusion

A series of S-substituted dithiocarbazate Schiff bases containing enantiomeric monoterpenes have been synthesized and their molecular structures were proposed on the basis of elemental analysis and various spectroscopic techniques. To the best of our knowledge, this is the first test of this class of compounds against clinically isolated DENV 2. Compounds have demonstrated strong antiviral potential of varying degree. Secondary evaluation shows that camphor containing compounds may have enhanced potential and should warrant further study.

## Supplementary material

IR spectra, EI-MS spectra, 1H and 13C{1H} spectra for compound 1–12 are provided in an attached supplementary document. Cambridge Crystallographic Data Centre (CCDC) 1947807–1947809 contains the supplementary crystallographic data for compounds 2, 4, and 5. These data can be obtained free of charge from the Cambridge Crystallographic Data Centre through www.ccdc.cam.ac.uk/data_request/cif.
